# The Epigenetic Regulation of Nonhistone Proteins by SETD7: New Targets in Cancer

**DOI:** 10.3389/fgene.2022.918509

**Published:** 2022-06-22

**Authors:** Chengyao Chiang, Heng Yang, Lizhi Zhu, Chunlan Chen, Cheng Chen, You Zuo, Duo Zheng

**Affiliations:** ^1^ Southern University of Science and Technology, Yantian Hospital, Shenzhen, China; ^2^ Guangdong Provincial Key Laboratory of Regional Immunity and Diseases, Department of Cell Biology and Genetics, Department of Pharmacy, Shenzhen University International Cancer Center, School of Medicine, The First Affiliated Hospital of Shenzhen University, Shenzhen Second People’s Hospital (Shenzhen Institute of Translational Medicine), Shenzhen University, Shenzhen, China

**Keywords:** SETD7, epigentics, non-histone substrate, transcriptional factor, protein methylaiton

## Abstract

Epigenetic modifications are essential mechanism by which to ensure cell homeostasis. One such modification is lysine methylation of nonhistone proteins by SETD7, a mono-methyltransferase containing SET domains. SETD7 methylates over 30 proteins and is thus involved in various classical pathways. As such, SETD7 has been implicated in both the basic functions of normal tissues but also in several pathologies, such as cancers. In this review, we summarize the current knowledge of SETD7 substrates, especially transcriptional-related proteins and enzymes, and their putative roles upon SETD7-mediated methylation. We focus on the role of SETD7 in cancers, and speculate on the possible points of intervention and areas for future research.

## 1 Introduction

The alteration of the gene expression profile in somatic cells is the main cause of human diseases. Such alterations can be driven by DNA methylation, posttranscriptional modification (PTM) of proteins, and noncoding RNAs (Esteller, 2007)—otherwise known as epigenetic modifications. Of the various PTMs, phosphorylation and acetylation help to modulate kinase activity and signal transduction. Ubiquitination and sumoylation regulate protein stability, while methylation influences protein interactions, function, stability, activity, structure and subcellular location (Wang et al., 2017). Many nonhistone proteins are also methylated; for example, lysine (K) can be mono-, di- or tri-methylated, while arginine (R) can be mono- or di-methylated (Pek et al., 2012). We are aware of around more than 50 lysine methyltransferases, 20 lysine demethylases (Han et al., 2019) and 10 proteins arginine methyltransferases (Wu et al., 2021) that are involved in protein methylation, either as a “writer” (adding methyl groups), a “reader” (recognizing the methyl signal), or an “eraser” (removing methyl groups). These proteins regulate several biological processes in both health and disease contexts.

SET domain containing lysine methyltransferase 7 (SETD7) is a 40 kDa protein containing 366 amino acids that is responsible for transferring the monomethyl group to lysine of its substrates from cofactor S-adenosylmethionine (AdoMet) (Fick et al., 2016). Similar to most lysine methyltransferases, the SET domain is required for catalysis, with histidine 297 the critical site for its methyltransferase activity (Nishioka et al., 2002). The methylated lysine targeted by SETD7 usually follows after the consensus motif of [K/R]-[A/S/T] (Del Rizzo and Trievel, 2011). SETD7 contains three membrane occupation and recognition nexus (MORN) motifs in the N-terminal region, which likely mediate SETD7’s interaction with the plasma membrane when the protein is not in the nucleus (Bivona et al., 2006).

SETD7, also known as SET7/9, KIAA1717, or KMT7, was first identified as a histone H3-lysine 4-specific (H3K4) methyltransferase that changes the affinity between histone 3 and double-stranded DNA to regulate gene expression (Wang et al., 2001). Since then, other have showed that SETD7 depletion has little impact on H3K4 methylation status in certain circumstances (Ea and Baltimore, 2009; Gaughan et al., 2011; Lehnertz et al., 2011), implying a more critical role of SETD7 on nonhistone proteins. SETD7 can modify many substrates, including histones and nonhistone transcription factors, transcriptional coactivators, hormone receptors, DNA methyltransferases, and other histone methyltransferases. The role of SETD7 is determined by the function of its substrates. Indeed, more than a dozen SETD7 nonhistone substrates have now been discovered (Keating and El-Osta, 2013).

The fates of the proteins modified by SETD7-mediated lysine methylation are diverse. They range from nucleus to cytoplasm and are implicated in gene transactivation, signaling transduction and regulation of hemostasis. Here, we discuss the known SETD7 substrates and their putative roles when methylated by SETD7 in regulation of cell cycle, apoptosis and response to external stimulation in human cancers.

## 2 SETD7-MEDIATED Substrate Modifications and Their Role in Cancer

### 2.1 Cell Cycle and Apoptosis Regulation

Cell cycle progression and cell apoptosis are coupled intimately. These important decisions of cell proliferation or cell death are likely to be controlled by more than one signal and are necessary to ensure a proper cellular response. Some proteins can involve in both cell division and programmed cell death, such as p53, pRb, E2F, which are responsible for reacting cellular stresses and regulating checkpoint-associated proteins, including CDK2 (Engeland, 2018). Basically, cell cycle is regulated by checkpoints which link the cell cycle to apoptotic pathways and ensure that cell cycle events toward the correct order, otherwise initiating cell apoptosis. Data thus far, programmed cell death and cell cycle share common molecular mechanisms, which are modulated by SETD7 via its methyltransferase activity.

#### TP53 and SIRTUIN 1 (SIRT1)

TP53, a tumor suppressor regulating cell cycle and controlling cell fate, is highly frequent loss-of-function in most of cancers, which is facilitated to cancer progression (Blagih et al., 2020). The TP53 protein can directly binds with transcription factors, including Sp1, TBP and NF-Y, to suppress genes expression (Liebl and Hofmann, 2021). Additionally, TP53 influences CDK-cyclin interaction through up-regulation of its downstream genes, such as *CDKN1A* (encode p21^Cip1/Waf1^ protein), resulting in down-regulation of cell cycle-associated genes (Hu et al., 2021). On the other side, several pro-apoptotic BCL-2 family members, including *BAX*, *BBC3* (*PUMA*) and *PMAIP1* (*NOXA*) are activated by TP 53, which leads to cell apoptosis (Parrales and Iwakuma, 2015). SETD7-mediated methylation of TP53 K372 potentiates apoptosis and facilitates the transcriptional initiation of TP53-downstream genes p21^Cip1/Waf1^ (Chuikov et al., 2004), to decelerate cell cycle progression. Others showed that murine TP53 K369 is also a potential target residue for methylation by SETD7. Methylation of this residue promotes TP53 acetylation by Tip60 and potentiates the expression of downstream genes, including p21^Cip1/Waf1^ and PUMA, *in vivo* (Kurash et al., 2008; Campaner et al., 2011). Methylation-dependent TP53 activation indicates a tumor suppressor role for SETD7 in cancer cells in both humans and mice.

Some epigenetic modifiers of TP53 are also reported as a substrate of SETD7, such as SIRT1 which is a nicotinamide adenine dinucleotide-dependent deacetylase, involving in various cell metabolic processes (Chen et al., 2021). SIRT1 is generally considered as an oncoproteins in leukemia and prostate cancer due to suppressing several tumor suppressors, such as TP53, via its deacetylase activity (Yousafzai et al., 2021). However, SIRT1-mediated regulation of TP53 is inhibited by SETD7-dependent methylation at K233, K235, K236 and K238 on SIRT1. However, multi-methylation does not influence SIRT1 deacetylase activity, which may induce a conformational change of SIRT1 to avoid TP53 binding (Liu et al., 2011). In addition to showing that SETD7 directly methylates TP53, the researchers showed an alternative way in which the transactivation capacity of TP53 can be enhanced during the DNA damage response. Taken together, SETD7 serves as a tumor suppressor to enhance TP53 activity by a novel manner through abolishment of SIRT1 and TP53 interaction.

#### E2 Promoter-Binding Factor 1 (E2F1) and Retinoblastoma Tumour Suppressor Protein (pRB)

E2F1 is a transcription factor responsible for the expression of DNA damage-induced genes, such as *CCNE1* which accelerates DNA replication and progression from the G1 to S phase of the cell cycle (Fouad et al., 2020). E2F1 also up-regulates downstream pro-apoptotic genes, including *TP73*, and activates programmed cell death through TP53-independent manner (Udayakumar et al., 2010). K185 on E2F1 is methylated by SETD7, which prevents E2F1 accumulation during DNA damage and activation of its proapoptotic target gene *TP73* via destabilization E2F1 by ubiquitination and degradation (Kontaki and Talianidis, 2010). However, other study reveals that SETD7 and LSD1 regulate E2F1-mediated apoptosis upon DNA damage. Methylation of K185 on E2F1 by SETD7 leads to E2F1 stabilization and up-regulation of proapoptotic genes *TP73* and *BIM*, whereas, SETD7-mediated effects are reversed by LSD1 (Xie et al., 2011). Interestingly, other study showed a negative correlation between E2F1 and SETD7 *in vivo* and in clinical specimens: Overexpression of E2F1 leads to SETD7 downregulation and EGFR and Snail upregulation in breast cancer cells (Montenegro et al., 2016). In the case as regulating its substrate, SETD7 is modulated by E2F1 either, which reveals a novel regulatory mechanism in SETD7 expression. Additionally, the threshold of expression of both E2F1 and SETD7 is indicated as a critical event to control the cell fate (Lezina et al., 2014). Since, the controversial role of E2F1 and its fully activity might also be determined the ubiquitinated level or types after SETD7-mediated methylation.

pRb functions in early cell cycle control by negatively regulating entry into S-phase by suppression of E2F1. In this way, pRb serves as a tumor suppressor, as well as usually being functionally inactivated in retinoblastoma, osteosarcoma, lung, breast and hepatic cancers (Giacinti and Giordano, 2006). Growth control by pRb is influenced by CDK phosphorylation, in which serial phosphorylation events that drive cell cycle transitions regulate pRb-dependent cell cycle progression (Mandigo et al., 2022). SETD7-mediated pRb methylation at K873 is required for pRb-dependent cell cycle arrest, transcriptional repression and pRb-dependent differentiation possibly by enhancing the interaction between pRb and the heterochromatin protein HP1 (Munro et al., 2010). The same group also demonstrated a novel mechanism in the regulation of E2F1 transactivation in which K810 methylation on pRb by SETD7 is essential for impeding cyclin/CDK recognition and the subsequent phosphorylation of the associated serine residue. As a result, pRb remains in the hypophosphorylated, growth-suppressing state (Carr et al., 2011). These data suggest that SETD7 serves as a tumor suppressor and cooperates with pRb in cell cycle control.

#### Forkhead Box O3 (FOXO3)

FOXO transcription factors have a critical role in longevity, tumor suppression and oxidative stress-induced neuronal cell death by regulating the expression of various target genes (Fasano et al., 2019). Activation of FOXO3 induces cell cycle arrest and promotes apoptosis in gastric cancer (Li M. et al., 2020), and pancreatic cancer (Usami et al., 2020). Other study shows that FOXO3 interacts with ERα and inhibits its transcriptional activity to suppress breast cancer progression (Zou et al., 2008). In addition, low expression of FOXO3 is associated with poorly prognostic outcome in estrogen-dependent breast cancer (Yin et al., 2020) and colorectal cancers (Bullock et al., 2013). FOXO3-mediated transcription and oxidative stress-induced neuronal apoptosis are negatively regulated by SETD7-dependent K270 methylation, as well as downregulating proapoptotic genes *BIM* (Xie et al., 2012). Interestingly, others showed that K271 on FOXO3 was methylated by SETD7, which decreases FOXO3 protein stability while moderately enhancing FOXO3-dependent activation of pro-apoptotic genes, which may in turn affect FOXO3’s ability to promote tumor suppression (Calnan et al., 2012). The role of SETD7 in methylation of K270 and K271 of FOXO3 is opposite and the detail molecular mechanism is demanded more evidences to clarify.

**FIGURE 1:**
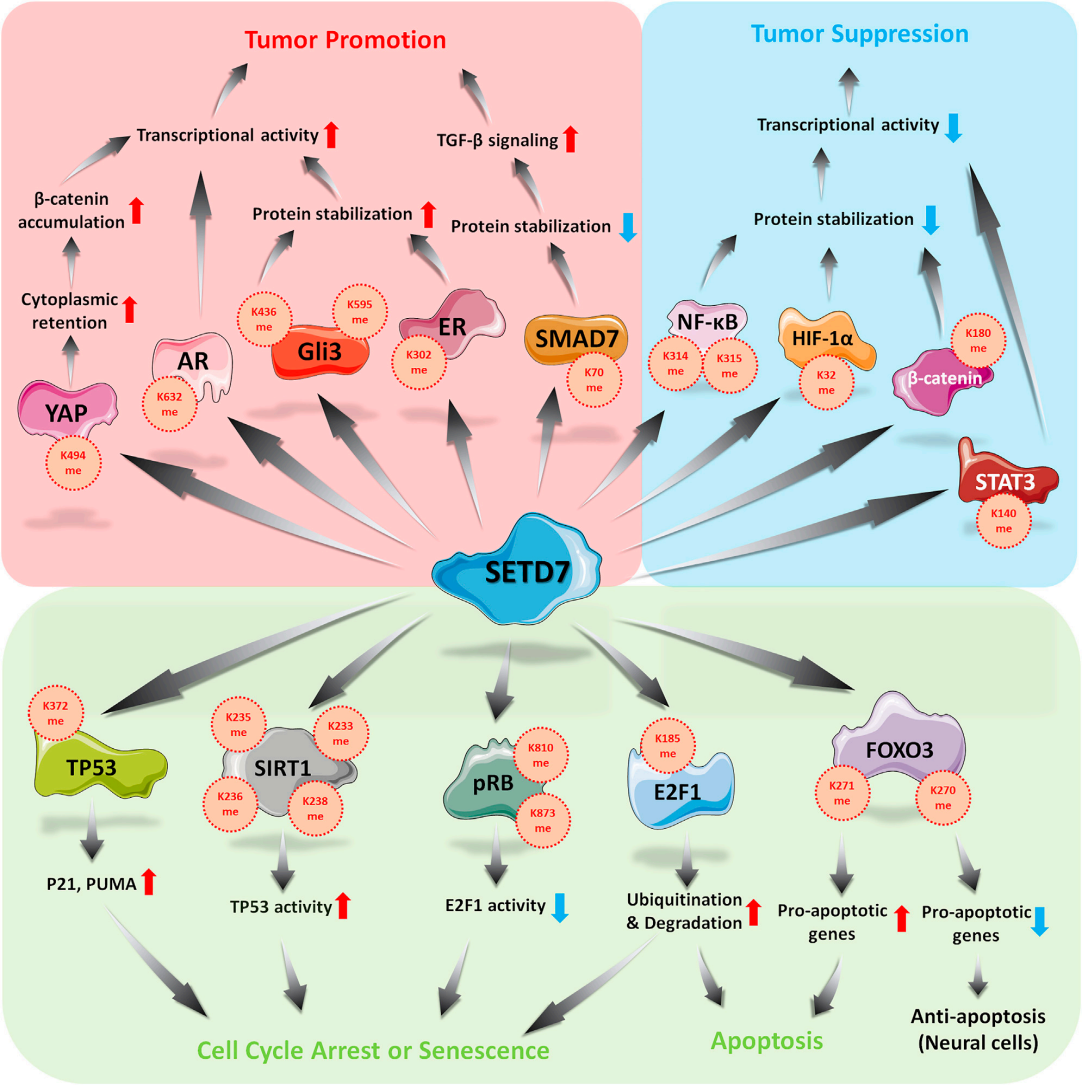
Roles of SETD7 in regulation of cell cycle, apoptosis and external stimulations.

The functions of SETD7 to its substrates and its effects in cell cycle and apoptosis regulation was summarized in Figure 1. For some controversial substrates, such as E2F1, FOXO3, whether tissue-specific interacting proteins or cooperation of other epigenetic modifications involved in SETD7-mediated regulation are such interesting issues, which is worth for further investigation in order to elucidate the exactly physiological effects of SETD7-substrates axis.

### 2.2 Regulation of External Stimulation

The corresponding responses of cells to various stimuli from micro-environment are essential strategies to homeostasis. Generally, transcription factors-mediated genes expression is responsible for implementing such reactions, for example, HIF-1α is activated by hypoxia stress and up-regulates *VEGF* (encode vascular endothelial growth factor) and *EPO* (encode erythropoietin) to overcome hypoxic condition (Plastino et al., 2021). Besides, cellular factors, such as cytokines, activate their corresponding receptors and downstream transcription factors or regulators, including SMADs (Zhang T. et al., 2020), STATs (Verhoeven et al., 2020), or NFκB (Zinatizadeh et al., 2021). Once such cellular responses might play pathological roles if the modulatory mechanisms are dysfunctional, as well as SETD7 showing its significant part among these regulatory processes.

#### Yes-Associated Protein and Beta-Catenin

YAP, a transcriptional co-activator belonged to Hippo pathway, is required for the growth of embryonic tissues, wound healing, and organ regeneration (Zanconato et al., 2016b). Activated YAP translocates into the nucleus and cooperates with transcriptional co-activator PDZ-binding motif (TAZ) to up-regulate proliferative- and anti-apoptotic-related genes, which is regulated by cell-intrinsic and -extrinsic signals, such as oxidative stress or nutrient-depletion (Koo and Guan, 2018; Moya and Halder, 2019). Unsurprisingly, YAP is hyperactivated in human malignancies (Moroishi et al., 2015), which can reprogram cancer cells into cancer stem cells and promote tumor initiation, progression and metastasis (Nguyen and Yi, 2019). Thus, YAP is emerging as a potentially therapeutic target for clinical application (Zanconato et al., 2016a). The monomethylation of K494 on YAP by SETD7 is critical for YAP cytoplasmic retention. This event thus represents a methylation-dependent checkpoint in the Hippo pathway (Oudhoff et al., 2013). As such, SETD7-dependent methylation of YAP facilitates Wnt-induced nuclear accumulation of β-catenin, linking the Wnt/β-catenin and Hippo/YAP pathways during intestinal regeneration and tumorigenesis (Oudhoff et al., 2016). These data indicate the triple layered regulation and crosstalk of two signaling pathways in an intestinal model.

On the other hand, β-catenin, a positive regulator in the canonical Wnt signaling pathway (Zhang and Wang, 2020), is activated by Wnt protein binding with frizzled receptors and translocates into the nucleus to participate in the transactivated complex (Cheng et al., 2019), promoting cancer progression by upregulation of proliferative-related genes (Zhang and Wang, 2020). Absence of Wnt stimulation, β-catenin is strictly modulated by glycogen synthase kinase-3 beta (GSK-3β) via S33 and S37 phosphorylation, which is recognized by E3 ligase β-TrCP, leading to ubiquitin-dependent proteosomal degradation (Valenta et al., 2012). Accumulating evidences refer oncogenic role of β-catenin in multiple human cancers, including solid tumors and hematological malignancies (Clevers and Nusse, 2012). Under condition of oxidative stress, SETD7 interacts with β-catenin and methylates it at K180, which promotes its phosphorylation by GSK3-β and subsequent degradation. The result is the suppression of downstream c-myc and cyclin D1 and inhibition of cell proliferation (Shen et al., 2015). However, this model currently lacks corresponding animal model and supportive clinical evidence. SETD7 is indeed emerging as a negative regulator of the Wnt/β-catenin pathway depending on the tissue or physical context. Notably, increasing epigenetic modifications on β-catenin is illustrated (Valenta et al., 2012), thus, it is interesting and necessary to be addressed that various of modifications regulate the same protein in certain physiological status.

#### NF-κB

NF-κB, a critical transcription factor in broad range of physiological functions, including inflammation, cell growth and programmed cell death, mainly retains in cytoplasm. Activated NF-κB, formed by RelA (also called p65) and p50 subunit (Zinatizadeh et al., 2021), translocates into the nucleus and up-regulates target genes after diverse extracellular stimuli, including TNF-α (Hoesel and Schmid, 2013), which might benefit for cell proliferation and survival in leukemia, melanoma, liver, breast, prostate and colorectal cancers (Dolcet et al., 2005). K314 and K315 on RelA can be methylated by SETD7, leading to destabilization of RelA in a ubiquitination-mediated manner (Yang et al., 2009), which results in downregulation of tumor-associated genes, such as IL-6, IL-8 and NOS-2. A contradictory role of SETD7 in the NF-κB pathway has also been described in which K37 on RelA was methylated, leading to stabilization of the RelA-DNA complex and enhanced expression of NF-κB-regulated genes (Ea and Baltimore, 2009). Similar results were also shown in diabetes models in which SETD7 interacts with RelA, facilitating the nuclear translocation of RelA and promoted function of NF-κB to transactivate downstream genes (Fujimaki et al., 2015; Chokpaisarn et al., 2017). NF-κB regulation by SETD7 might depend on the cellular context, tissue specificity or particular physiological condition, such as in cancer cells or diabetes model.

#### Hypoxia Inducible Factor

Hypoxia inducible factor-1α (HIF-1α) is a transcription factor involved in adaption of low oxygen concentration. Under normoxia, HIF-1α is strictly modulated by an E3 ligase von Hippel-Lindau (VHL), which induces ubiquitination-dependent proteasomal degradation of HIF-1α (Semenza, 2003). Activated HIF-1α translocates into the nucleus and activates targeting genes, which participates in tumor angiogenesis, metastasis, invasion and glucose homeostasis in various cancer cell lines (Elzakra and Kim, 2021; Satija et al., 2021). Here, K32 methylation of HIF-1α by SETD7 promotes HIF-1α degradation in the nucleus and thus the inhibited expression of downstream genes in a proline hydroxylation-independent manner. This effect can be restored upon exposure to the demethylase LSD1 (Kim et al., 2016). Both HIF-1α and HIF-2α are reported substrates of SETD7 and are methylated on K32 and K29, respectively, due to their homologous of sequence. Interestingly, *SETD7* expression is suppressed under hypoxic conditions (Liu et al., 2015). Others have reported that SETD7 is a negative regulator of HIF-1α and downregulates HIF-1α target genes, such as *GLUT1, LDHA, PGK1, EPO, PKM2* and *VEGF,* which are upregulated after SETD7 inhibition (Li et al., 2021; Xiaoshi et al., 2021).

#### Esrtogen Receptor and Androgen Receptor

ER and AR, ligand-dependent transcription factors, are activated by sex hormones and responsible for the regulation of cell proliferation, survival and differentiation (Shafi et al., 2013; Berkel and Cacan, 2021) in breast (Anestis et al., 2020) and prostate cancer (Tan et al., 2015), respectively. As most transcription factors, activated ER or AR translocates into the nucleus and recruits other epigenetic enzymes, such as histone acetyltransferase or methyltransferase, to transactivate targeting genes expression (Waddell et al., 2021). Unsurprisingly, aberrant expression ER and AR are risk factors in many cancers, including prostate, breast and lung cancers (Burstein, 2020). Anti-ER or AR approaches thus seem as effective options for such type of cancers. Here, SETD7-mediated methylation of K302 on ER ensures protein stability and promotes DNA binding activity and the expression of ER-downstream genes, such as *PS2* and progesterone receptor (*PgR*), in breast cancer. These results imply that lysine methylation of ER facilitates to prevent ubiquitination on the same residue by E3 ligases (Subramanian et al., 2008). Similarly, SETD7 interacts directly with AR and enhances AR transcriptional activity by methylating its K632 residue (Gaughan et al., 2011), which is not only plays a proliferative role in prostate cancer but is also involved in TNFR and PTEN/PI3K/AKT signaling (Wang et al., 2018). SETD7 thus seems to be a coactivator of hormone receptors, and in this way helps to promote carcinogenesis. Therefore, ER or AR combines with SETD7 might serve as the panel of prognostic markers or therapeutic targets for patients with such cancers.

#### Gloma-Associated Oncogene Homolog

GLIs, a family of zinc finger transcription factors, serve as nuclear mediators of the Hedgehog pathway and regulate genes essential for various stages of tumor development and progression (Naruse et al., 2010; Katoh, 2019). Without ligand stimulation, GLIs are suppressed by suppressor of fused (SUFU), leading to cytoplasmic retention (Sasai et al., 2019). Aberrant activation of Hedgehog-GLI axis is reported in human malignancies, including breast, pancreatic, lung and ovarian cancers, which resulted in upregulation of oncogenic genes (Matissek and Elsawa, 2020), such as *BCL2*, *CCND1*, *MYCN*, *NANOG*, *SOX2* and *SNAL1*. As such, GLI family members might be therapeutic targets in various cancers (Niewiadomski et al., 2019). In previous study, GLI3 K436 and K595 residues are methylated by SETD7, which stabilizes GLI3 protein and in turn activates the Sonic Hedgehog pathway, resulting in the expression of downstream genes, including *Ptch1.* These genes promote proliferation, invasion and metastasis of non-small-cell lung cancer cells (Fu et al., 2016). To date, however, a clinical correlation between SETD7 and GLI3 expression at protein level is lacking, which is still fuzzy to figure out the significance of GLI3-dependent oncogenesis by SETD7.

#### Small Mothers Against Decapentaplegic

SMADs, critical regulators participated in transforming growth factor-beta (TGF-β) signaling, have key roles in development, carcinogenesis and fibrogenesis (Derynck and Zhang, 2003). After receptor activating, SMAD2, 3, and 4 translocate into the nucleus to turn on oncogenic genes, including *SNAIL* and *SLUG* (Zhang T. et al., 2020). While SMAD7 serves as a negative modulator to promote degradation of TGF-β receptor by recruiting E3 ligase SMURF1/2 (Smad ubiquitin-related factor1/2) (Colak and Ten Dijke, 2017). Epigenetic modification on K70 of SMAD7 by SETD7-mediated methylation decreases the protein stability of SMAD7 by ubiquitination-dependent manner via Arkadia E3 ligase in mouse models of pulmonary fibrosis. In SETD7-deficient mice, TGF-β-induced lung fibrosis is highly ameliorated (Elkouris et al., 2016), which indicates SETD7 is a positive regulator in TGF-β signaling, even plays an oncogenic role in TGF-β-mediated cancers, such as breast cancer and glioblastoma (Colak and Ten Dijke, 2017). As such, SETD7 might be a potential therapeutic target for lung fibrosis or cancers. Others reported that SETD7 interacts with SMAD3, but not SMAD2, to ensure protein stability, which is beneficial for increasing of collagen contractility, as well as wound healing in renal fibroblast (Shuttleworth et al., 2018). The effects of SETD7 on various SMAD proteins thus seem to be diverse, which is determined by unique sequence and structure of each protein, or interacting proteins in the same protein family.

#### Signal Transducer and Activator of Transcription 3

STATs, a family of cytoplasmic transcription factors shared an overall general structure, are responsible for responding to cytokine stimulation (Bose et al., 2020). Among them, STAT3 is involved in numerous biological processes, including cell proliferation, survival, differentiation, and angiogenesis (Xin et al., 2020). STAT3 is hyperactivated in most human cancers, such as prostate, breast, and ovarian cancer (Yu et al., 2014), and is generally associated with a poor clinical prognosis (Zou et al., 2020). Previous study showed that tyrosine phosphorylation is an essential event for K140 methylation on STAT3 by SETD7. Moreover, STAT3 activity and its target gene expression are partially repressed by SETD7-mediated STAT3 methylation when IL-6 stimulation (Yang et al., 2010). In this case, SETD7 seems to serve as both an inflammatory and tumor suppressor.

SETD7 exhibits it impact as either an oncogenic protein or a tumor suppressor (Figure 1), thus, SETD7 is emerging as a therapeutic target in YAP-, ER-, AR-, and GLI3-mediated tumorgenesis. Recently, (*R*)-PFI-2 was identified as a first-in-class, potent (*Ki* ^*app*^ = 0.33 nM), selective, and cell-active inhibitor of the methyltransferase activity of human SETD7 (Barsyte-Lovejoy et al., 2014). (*R*)-PFI-2 exhibits an unusual cofactor-dependent and substrate-competitive inhibitory mechanism by occupying the substrate peptide binding pocket of SETD7, including the catalytic lysine-binding channel, and by making direct contact with AdoMet (Lenstra et al., 2018). (*R*)-PFI-2 showed its activity in breast cancer cell MCF7 and mouse embryonic fibroblast in YAP-related studies (Barsyte-Lovejoy et al., 2014) and thus might be a potential therapeutic option for SETD7-mediated disease progression.

## 3 Perspectives and Concluding Remarks

Methylation events serve to modulate and fine tune various cellular processes and signaling pathways (Han et al., 2019). As we have outlined in this review, SETD7-mediated methylation of transcription-related factors and enzymes (Table 1) has wide-reaching effects in different cell types and contexts. For example, SETD7 may act as either an oncogene or tumor suppressor. Meanwhile, SETD7-mediated methylation at different lysine residues within the same protein can even lead to divergent outcomes in different cancer cells and contexts (Batista and Helguero, 2018). Given the potential implications of intervening on SETD7-mediated methylation in disease contexts, namely cancer, researchers are keen to discover novel SETD7 substrates. Currently, researchers can use online prediction software of putative methylation sites combined with the SETD7 consensus methylation sequence to explore uncharacterized candidate proteins or isoforms of known SETD7 substrates, as exemplified for HIF-1α and HIF-2α (Liu et al., 2015).

**TABLE 1:** SETD7-regulated proteins and methylation sites.

Substrate	Methylation Site	Sequence Around Methylation Site											Study Model	Consequence	Role of SETD7	References
		−5	−4	−3	−2	−1	0	1	2	3	4	5				
Transcriptional-related factors																
TP53	K372	S	H	L	K	S	K	K	G	Q	S	T	293F, U2OS (Osteosarcoma), H1299 (NSCLC)	Enhancement of transactivation	Tumor suppressor	Chuikov et al. (2004)
NF-κB (Rel A)	K314, K315	F	K	S	I	M	K	K	S	P	F	S	MEFs (Mouse embryonic fibroblast), U2OS (Osteosarcoma), A549 (NSCLC)	Protein degradation	Tumor suppressor	Yang et al. (2009)
	K37	M	R	F	R	Y	K	C	E	G	R	S	HEK293T	Stabilization of RelA-DNA complex	Oncoprotein	Ea and Baltimore, (2009)
HIF-1α	K32	R	S	R	R	S	K	E	S	E	V	F	Hela (Cervical cancer), RCC4 (Kidney cancer)	Protein degradation	Tumor suppressor	Kim et al. (2016)
HIF-2α	K29	R	C	R	R	S	K	E	T	E	V	F	RCC4 (Kidney cancer)	Suppression of transactivation	Tumor suppressor	Liu et al. (2015)
ER	K302	M	I	K	R	S	K	K	N	S	L	A	Breast cancer	Protein stabilization	Oncoprotein	Subramanian et al. (2008)
AR	K632	G	A	R	K	L	K	K	L	G	N	L	Prostate cancer	Enhancement of transactivation	Oncoprotein	Gaughan et al. (2011)
Gli3	K436	H	N	K	R	S	K	I	K	P	D	E	NSCLC	Protein stabilization	Oncoprotein	Fu et al. (2016)
	K595	H	E	G	C	N	K	A	F	S	N	A				
E2F1	K185	I	A	K	K	S	K	N	H	I	Q	W	NSCLC	Protein stabilization/degradation	Controversial	Lezina et al. (2016)
β-catenin	K180	V	H	Q	L	S	K	K	E	A	S	R	Hela (Cervical cancer)	Protein degradation	Tumor suppressor	Shen et al. (2015)
SMAD7	K70	A	V	R	G	A	K	G	H	H	H	P	Lung fibroblasts, Hela (Cervical cancer)	Protein degradation	Fibrosis suppressor	Elkouris et al. (2016)
YAP	K494	V	L	A	A	T	K	L	D	K	E	S	Mice intestinal tumor	Cytoplasmic retention	Oncoprotein	Oudhoff et al. (2013)
TAF10	K189	S	R	S	K	S	K	D	R	K	Y	T	HEK293, F9 Embryonic carcinoma	Enhancement of TAF10-RNA polymerase II complex	Controversial	Kouskouti et al, (2004)
FOXO3	K270	G	R	A	A	K	K	K	A	A	L	Q	HEK293T	Protein degradation	Neural apoptosis suppressor	Xie et al. (2012)
	K271	R	A	A	K	K	K	A	A	L	Q	A	HEK293T, NIH-3T3	Protein degradation/Moderately enhancement of transactivation	Tumor suppressor	Calnan et al. (2012)
STAT3	K140	A	V	V	T	E	K	Q	Q	M	L	E	DLD1(Colon cancer)	Partial repression of transactivation	Tumor suppressor	Yang et al. (2010)
SOX2	K42	S	P	D	R	V	K	R	P	M	N	A	PA-1 (Ovarian teratocarcinoma)	Protein degradation	Tumor suppressor	Zhang et al, (2018)
	K117	P	R	R	K	T	K	T	L	M	K	K				
pRb	K810	Y	I	S	P	L	K	S	P	Y	K	I	Hela (Cervical cancer), CC42 (Mouse B cell hybridoma), C2C12 (Mouse myoblast), U2OS and SAOS2 (Osteosarcoma)	Protein stabilization	Tumor suppressor	Carr et al. (2011)
	K873	P	P	K	P	L	K	K	L	R	F	D				Munro et al. (2010)
**Substrate**	**Methylation Site**	**Sequence Around Methylation Site**											**Study Model**	**Consequence**	**Role of SETD7**	**References**
		**−5**	**−4**	**−3**	**−2**	**−1**	**0**	**1**	**2**	**3**	**4**	**5**				
Enzymes																
SUV39H1	K105	R	H	H	R	S	K	T	P	R	H	L	MEFs (Mouse embryonic fibroblast), H1299 (NSCLC)	Inhibition of enzyme activity	Tumor suppressor	Wang et al. (2013)
	K123	L	V	Q	K	A	K	Q	R	R	A	L				
ARTD1/PARP1	K508	L	S	K	K	S	K	G	Q	V	K	E	U2OS (Osteosarcoma), MEFs (Mouse embryonic fibroblast)	Facilitation of DNA repair	Oncoprotein	Kassner et al. (2013)
RIOK1	K411	A	S	Q	R	T	K	E	E	R	S	S	Colorectal and gastric cancers	Protein degradation	Tumor suppressor	Hong et al., 2018
SIRT1	K233	L	S	E	P	P	K	R	K	K	R	K	HEK293T, HCT116 (Colorectal cancer)	Interaction with p53	Tumor suppressor	Liu et al., 2010
	K235	E	P	P	K	R	K	K	R	K	D	I				
	K236	P	P	K	R	K	K	R	K	D	I	N				
	K238	K	R	K	K	R	K	D	I	N	T	I				
PCAF	K89	S	A	P	R	A	K	K	L	E	K	L	HEK293, U2OS (Osteosarcoma)	Nuclear localization	Controversial	Masatsugu and Yamamoto, (2009)
DNMT	K142	T	P	R	R	S	K	S	D	G	E	A	Breast cancer	Protein degradation	Tumor suppressor	Esteve et al. (2009)

Gene expression depends on not only activity of transcription factors, but also heterochromatin status which is regulated by some epigenetic modifiers. Besides SIRT1 we mentioned before, SETD7-mediated methylation has significance in regulation of such modifiers, such as suppressor of variegation 3-9 homolog 1 (SUV39H1) (Wang et al., 2013), p300/CBP-associated factor (PCAF) (Masatsugu and Yamamoto, 2009), ADP-ribosyltransferase diphtheria toxin-like 1 (ARTD1/PARP1) (Kassner et al., 2013) and DNA methyltransferase (DNMT) (Esteve et al., 2009). A multi-layered and -dimension regulatory network of SETD7 reveals the complexity and diversity of genetic modulation in the nucleus.

Data thus far, however, suggest that SETD7 exhibits a suppressive pattern in breast cancer, having a negative correlation with DNMT and E2F1 expression (Montenegro et al., 2016). In addition, low SETD7 expression correlates with a poor prognosis and lower survival rate in patients with gastric cancer (Akiyama et al., 2016), colorectal cancer (Zhang S. L. et al., 2020) and glioma metastasis (Li C. et al., 2020). On the other hand, data from a cohort study showed a positive correlation between SETD7 expression and the staging of cancer progression, which also seems to serve as a serum biomarker in colorectal cancer (Duan et al., 2018). SETD7 is also reported to have an oncogenic character in hepatoma cellular carcinoma, being progressively upregulated according to cancer stage (Gu et al., 2018). Interestingly, strong nuclear staining of SETD7 in high grade patients suggests that its subcellular localization is a significant indicator in the development and progression of prostate cancer (Gaughan et al., 2011), which might associate with the role of AR in nucleus. Due to the complexity of clinical specimens and differences among individuals, verifying the role of SETD7 and its corresponding substrates is unlikely in most cancer types. For this reason, the study of SETD7 is still largely confined to cellular based research or studies conducted in animal models.

Remarkably, SETD7 also acts as a tumor suppressor in certain contexts and indeed is downregulated in some cancers; thus, a method by which to elevate SETD7 expression and increase its activity is also warranted. Berberine, an anticancer agent, is a major botanical alkaloid that can be isolated from the root of *Rhizoma coptidis* (Huanglian) (Khan et al., 2022). Berberine can modulate various methylation- and acetylation-related enzymes that upregulate SETD7 expression in human multiple myeloma U266 cells in a dose-dependent manner (Wang et al., 2016). Moreover, SETD7 upregulation by berberine promotes RelA methylation and suppresses RelA-dependent transactivation of miR-21 in U266 cells (Hu et al., 2013). Unfortunately, due to the multi-bioactivity of berberine, a more specific agonist or inducer needs to be discovered in order to avoid off-target effects.

Going forward, further studies into the physiological and pathological effects of SETD7 are warranted to help develop novel diagnostic, prognostic, and/or therapeutic approaches in the cancer contexts. Although not discussed in this review, SETD7 is also a potential target to ameliorate diabetes, inflammatory diseases, and aging-associated disorders (Batista and Helguero, 2018). However, as more and more substrates of SETD7 are discovered, researchers have to consider the effects from known substrate of SETD7 in their models when they find a novel candidate of SETD7, which might be as a reason leading to the decreasing of SETD7-related articles in recent years. According to tissue or cellular specificity, SETD7-related studies tend to investigate multi-substrate interactions in the same model to determine the ultimate effects of increasing or reducing various factors. Improving our fundamental knowledge on the mechanism of SETD7-mediated regulation of its substrates will be extremely informative to define tissue and cellular characteristics that are beneficial for SETD7-associated therapies.
